# A qualitative study of patient experiences and expectations around hospital care during exacerbations of chronic obstructive pulmonary disease – a health CASCADE study

**DOI:** 10.1186/s12890-025-04024-x

**Published:** 2025-11-13

**Authors:** Qingfan An, Marlene Sandlund, Margrit Schreier, Karin Wadell, Sara Lundell

**Affiliations:** 1 Department of Community Medicine and Rehabilitation, Physiotherapy, Umeå University, Umeå, SE-901 87 Sweden; 2 School of Business, Constructor University, Bremen, Germany

**Keywords:** Pulmonary disease, chronic obstructive, Hospitalisation, Qualitative, Patient experience, Patient expectation

## Abstract

**Introduction:**

Hospital care is critical when assessing the overall quality of chronic obstructive pulmonary disease (COPD) care, particularly for people living with severe COPD who are frequently hospitalised due to exacerbations. The hospitalisation experience for people with COPD is often complex, involving various interactions and has been reported to be suboptimal. A comprehensive understanding of these experiences is lacking. With the intention of informing a holistic approach in COPD hospital care, this study aimed to explore the experiences and expectations of individuals with severe COPD regarding hospital care due to exacerbations of COPD.

**Methods:**

Acknowledging the complexity of interactions within the studied scenarios, this research employed a qualitative study design, utilising co-creation workshops for data collection. A total of 13 participants were involved in the data collection process. Five people with severe COPD were recruited using purposive sampling. In addition, one family member, four healthcare practitioners, one digital health program designer, and two hospital managers were recruited through convenience sampling. Data were collected during three co-creation workshops. During each workshop, participants were divided into subgroups focused on specific topics. Relevant transcripts from these subgroup discussions were chosen for analysis, which was conducted using qualitative content analysis.

**Results:**

The analysis resulted in four categories that illustrate both the experiences and expectations of people with COPD regarding hospital care from admission to discharge: lack of trustworthy guidance, increased vulnerability during hospitalisation, discharge issues, and advocacy for COPD recognition. Our findings reveal some deficiencies, particularly in admission procedures, information exchange, healthcare interactions, and transitions from hospital to home. At the same time, patients also expressed appreciation for the continuous support provided by COPD nurses and home care teams.

**Conclusion:**

This study highlights the need for person-centred care in managing COPD exacerbations. It identifies key interventions such as early help-seeking, better patient education, staff training, care continuity, improved discharge services, and public awareness. Emphasising individualised experiences, it calls for familiar care settings, collaborative discharge planning, and integration of home care to enhance hospital care quality.

**Supplementary Information:**

The online version contains supplementary material available at 10.1186/s12890-025-04024-x.

## Background

 Chronic obstructive pulmonary disease (COPD) is a common, preventable and treatable disease, now counted as the fourth most common cause of death globally [1, 2]. People with COPD complain of dyspnea and activity limitation and most people with COPD have several comorbidities [3], complicating their care. Exacerbations of chronic obstructive pulmonary disease (ECOPD), sometimes referred to as ‘lung attacks’, worsen health outcomes, hospitalisation rates, disease progression, and mortality [2]. About 25% of patients hospitalised for an exacerbation die within a year, and up to 65% within five years [4]. Due to frequent rehospitalisations, ECOPD significantly contributes to the financial burden on society [5–7]. Remarkably, one-fifth of people with COPD are readmitted within 30 days post-discharge [8], resulting in unexpectedly high costs for the healthcare system and posing additional challenges for patients and their families [7, 9].

Despite these costs, hospitalisation experiences for patients are often suboptimal, compounded by challenges faced by healthcare practitioners such as limited COPD knowledge and high workloads [10–12]. Positive patient experiences are linked to improved mortality, morbidity, and medication adherence [13]. Existing studies often concentrate on isolated aspects of COPD hospitalisation when assessing patient experiences, such as treatment, symptom management, and staff interactions [10]. This fragmented exploration may restrict the broader understanding necessary to adopt a more holistic approach to improving care quality [10]. Efforts to improve hospital care ought to move beyond the inpatient stay to encompass the entire care continuum, including admission, discharge planning, and post-discharge support, as all stages shape the patient’s overall hospital experience.

Understanding patients’ experiences and expectations as care recipients is essential to comprehensively explore the hospitalisation journey, identify opportunities to improve care quality, and inform person-centred care efforts [14, 15]. These insights play a crucial role in improving the quality of care delivered by healthcare practitioners across all levels [10]. Exploring patient perspectives serves as a critical first step toward the future development of relevant guidelines and interventions. One way of exploring patient perspectives is to use participative methodologies, such as co-creation. Co-creation involves a diverse range of stakeholders who take part in the scenarios being studied, making it valuable in exploring complex healthcare settings [16]. While co-creation often involves smaller sample sizes to ensure meaningful participation, it provides high-quality insights by promoting equitable communication and facilitating discussions from the patients [17]. Therefore, the aim of this study was to explore the experiences and expectations of people with severe COPD regarding hospital care due to ECOPD.

## Methods

### Study design

This qualitative study employed co-creation workshops, adhering to the Consolidated Criteria for Reporting Qualitative Research (COREQ) guidelines [18]. Conducted in accordance with the Declaration of Helsinki, it was approved by the Swedish Ethical Review Authority (Dnr. 2022–03695−01). Signed informed consent was obtained from all participants. This analysis is a sub-study within a broader co-creation initiative aimed at co-creating interventions to improve the hospitalisation experiences of people with COPD.

Patient experiences and expectations emerged as a key focus in the larger initiative, and due to their significance, they were further explored in this separate analysis. This initiative is part of Health CASCADE, an EU-funded Marie Skłodowska-Curie Innovative Training Network, aiming to make co-creation trustworthy and effective for addressing public health problems, as detailed elsewhere [19].

Discussions initially focused on early discharge and related hospitalisation challenges [8], a contextually relevant issue that helped elicit patient experiences and expectations of hospital care. Generally, in Sweden, patients are advised to first consult 1177 Vårdguiden, the Swedish National Health Information Service, to determine whether self-management strategies can be applied [20]. The next level of care is primary care, which manages further assessment and treatment [20]. If more advanced care is needed, primary care providers refer patients to secondary (specialised) care or to the emergency department [20]. Challenges related to hospitalisation and early discharge often emerge within this pathway and are reflected across these different levels of care.

### Setting

The study involved participants from a hospital in northern Sweden, serving about 900,000 people. Co-creation activities were conducted at the UMeHealth Lab, a specialised research facility at Umeå University.

### Participants and recruitment

This study recruited 5 people with COPD using purposive sampling. Eligibility criteria are listed in Table 1. The characteristics of the people with COPD are presented in Table 2. Over the past year before recruitment, two patients had a total of four hospitalisations due to ECOPD. One of these patients also experienced more than five moderate exacerbations. Additionally, three other patients each had one hospitalisation due to ECOPD, with two of them also having more than five moderate exacerbations during this time. At the time of the study, no patient participant was receiving long-term oxygen therapy (LTOT) or non-invasive ventilation (NIV) at home. To complement and enrich patient perspectives, additional groups of stakeholders were recruited through convenience sampling, with the condition that they had experience in the treatment or care of individuals with COPD. A family member was included to provide insights into supporting and navigating the patient’s hospital journey. Four healthcare practitioners (nurse, physiotherapist, dietician, physician) and two hospital managers were included for their direct roles in hospital-based COPD care. A digital health program designer was included to explore if and how digital tools could support care across the hospital continuum. The family member was a female with a health literacy score of 302 and an eHealth literacy score of 19. Among the healthcare practitioners, designer, and hospital managers (5 females, 2 males, aged 25–62), their professional experience ranged from 0.8 to 34 years, with their specific experience working with COPD ranging from 0.8 to 23 years. Participants in the study were approached via phone calls for recruitment. All patients, the relative, and healthcare practitioners that were asked to participate agreed to participate in the study, except for one physician who declined due to workload.

**Table 1:** Eligibility criteria for recruiting people with COPD

Inclusion criteria	Diagnosed with COPD according to Global Initiative for Chronic Obstructive Lung Disease (GOLD) (2)
	Classified as GOLD stage 3 (FEV₁ 30–50% predicted) or GOLD stage 4 (FEV₁ <30% predicted) based on post-bronchodilator spirometry (2)
	Had experienced at least one hospitalisation due to ECOPD in the previous 6 months prior to recruitment (i.e. classified as risk group E according to GOLD (2))
	Stable disease state as defined as no exacerbation in the previous one month prior to recruitment
	The ability to read, write, and speak Swedish language
	Live in northern Sweden
	Regularly use tablet or smartphone
Exclusion criteria	Cognitive decline

**Table 2:** Characteristics of the COPD patients

Characteristics	COPD patients (*n* = 5)
Gender (w/m)	3/2
Age (years)	66–82
FEV1 predicted (%)	23–36
GOLD stage (*n*)	GOLD stage 3, risk group E (3) GOLD stage 4, risk group E (2)
COPD assessment test^a^ (min–max)	10–27
Length of last hospitalisation	1–5 days
Communicative and critical health literacy scale (Swedish version)^b^ [21] (min–max/median)	5–1301/104
Electronic health literacy scale (Swedish version)^c^ [22] (min–max/median)	12–39/32

*COPD* chronic obstructive pulmonary disease, *w* women, *m* men, *FEV1* Forced expiratory volume in one second, *GOLD* Global Initiative for Chronic Obstructive Lung Disease

^a^Scores ranging from 0 to 40, with higher values indicating worse symptoms

^b^Scores ranging from 5 to 5000, with higher values indicating lower health literacy

^c^Scores ranging from 8 to 40, with higher values indicating higher electronic health literacy

### Research team

The research group consisted of 5 researchers. Four researchers QA (PhD student, female), MS (PhD, female), KW (professor, female), and SL (PhD, female) participated in study design, data collection and data analysis, while one researcher MSch (professor, female) was only involved in the analysis process. QA prepared the materials used in the workshop, while MS, KW, and SL facilitated the workshops. QA drafted the first version of the manuscript, and all researchers were involved in revising the manuscript and approved the final version. QA has an industrial design background, while MS, KW, and SL are physiotherapists with experience of facilitation of co-creation workshops. KW and SL have experience in COPD research and KW has extensive clinical experience of the patient population. MS, MSch, KW and SL are all experienced in qualitative research. At the time of the study, QA was a PhD student, while MS and SL held positions as associate professors, and MSch and KW were professors. KW had patient-provider contact with certain individuals with COPD and was colleagues with some healthcare practitioners. KW and SL had been involved in earlier studies with the COPD nurse (designated nurses responsible for COPD care) and the designer.

### Data collection

Data for this qualitative study were collected during three two-hour co-creation workshops with varying participant numbers due to availability. Facilitated by MS, KW and SL, these workshops featured topic-specific subgroup discussions, with smaller groups engaging in focused conversations. All discussions were audio recorded. The facilitators took field notes during the sessions. At the beginning of the workshops, the researchers introduced themselves, their roles and the aim of the study. Only participants and researchers were present during the workshops. The materials (i.e. question guides) used to stimulate the discussions can be found in Appendix A. Following each workshop, the findings were compiled and presented at the subsequent session for member checking [23] to ensure accuracy and validation. The workshops and the description of the subgroup discussions included in the analysis are shown in Table 3. The transcripts of the subgroup discussion were included in the analysis only if patients were involved.

**Table 3:** Source and aims for the selected subgroup discussions included in the analysis

Workshop	Included subgroup discussion (*n* = 7)	Participants in the included subgroup discussions	Aim of the subgroup discussion
1st	No. 1	5 people with COPD	To explore the causes and consequences of early discharge for people with COPD.
2nd	No. 2 (Task 1)	1 family member, 1 hospital manager, 1 physiotherapist, and 1 person with COPD	To discuss their visions for hospital care in the year 2050, ensuring the integration of patient expectations with those of other stakeholders.
	No. 3 (Task 1)	1 COPD nurse, 1 hospital manager, 1 person with COPD, 1 dietician	
	No. 4 (Task 1)	1 designer working with 1177, 1 physician, 1 person with COPD	
	No. 5 (Task 2)	1 physiotherapist, 1 hospital manager, 1 dietician, 2 people with COPD, 1 COPD nurse,	To explore co-created futures in hospital and primary care centre/home/community settings if the problems are solved.
	No. 6 (Task 2)	1 designer working with 1177, 1 person with COPD, 1 family member, 1 hospital manager	
3rd	No. 7	5 people with COPD and 1 family member	To explore the emotional status, the unmet needs, and the viable solutions to the unmet needs during the hospitalisation process for patients.

#### The first co-creation workshop

The objective of the first workshop was to get a shared understanding among co-creators of the causes and consequences of early discharge from the hospital following an ECOPD. Participants were divided into three subgroups: one with allied healthcare practitioners and a family member, another with managers, a physician, and a designer, and the third with people with COPD. Each subgroup was tasked with identifying the causes and consequences of early discharge through subgroup discussions employing the world café method [24]. The problem was framed as: “People with COPD who are hospitalised for exacerbations are not always recommended or provided with sufficient healthcare interventions before being discharged home.”

#### The second co-creation workshop

The second workshop aimed to help participants articulate their shared ideal futures related to the hospitalisation process. Participants were initially divided into three mixed subgroups to discuss their visions for hospital care in the year 2050 (task 1), ensuring the integration of patient expectations with those of other stakeholders. Subsequently, participants were reshuffled into pairs to collectively envision their preferred future lifestyles in different settings, including hospital, primary care, home, and community. Creative brainstorming was facilitated using a deck of cards inspired by “The Thing from the Future” card game [25] to stimulate imaginative narratives of future sociotechnical systems related to COPD care. Subsequently, the pairs were reassembled into two larger groups to present their envisioned future lifestyles (task 2).

#### The third co-creation workshop

During the third workshop, participants were divided into three groups: one with people with COPD and a family member, another with a designer, physiotherapist, and COPD nurse, and the third with hospital managers. The primary objective of the patient group was to delve into emotional experiences, unmet needs, and potential solutions across various stages of the hospitalisation process, utilising the user journey map method [26]. This method outlined the user experience across various stages of the service, with specific stages tailored to the hospitalisation process for people with COPD [26]. Details on how we adapted and applied this method are reported elsewhere [27].

### Data analysis

Transcription was done by a specialised company, translating Swedish text to English, verified for accuracy by MS, KW, and SL. Qualitative content analysis, following Graneheim and Lundman’s approach, was primarily employed [28]. The units of analysis were the subgroup discussion transcripts (totalling 7). Content reflecting patient experience and expectations was segmented into meaning units, and only statements directly attributable to patients, or those made by other stakeholders that were not contested by the patients, were coded, ensuring fidelity to patient perspectives. Initial coding of meaning units was conducted by QA. During this process, post-it notes were used as participant-driven summaries of key priorities, aiding the coding effort. Subsequently, three additional researchers (MS, MSch and KW) reviewed and cross-checked the codes, engaging in discussions and providing occasional suggestions for further interpretations and codes. The coding process was supported by MAXQDA software (MAXQDA standard 2022, VERBI GmbH, Berlin, Germany). A classification process was done by researchers (QA, MS and SL) through comparing the codes based on their similarities and differences. Subcategories were formed, and categories were created by constantly comparing the subcategories. Analysis involved iterative examination of transcripts, codes, and subcategories to ensure the findings were grounded in empirical data. The created subcategories and categories were discussed among all authors. Examples illustrating the analysis process from raw quotes to codes, subcategories, and overarching categories are presented in Table 4.

**Table 4:** Illustrative examples of the analysis process

Example quote	Code	Subcategory	Category
“But you worry that ‘will it get even worse? Is it going to be a hospital again?’” (Workshop 4, Patient 5) “Maybe it gets better. That’s my experience.” (Workshop 4, Relative) “Yes. Hold out as long as possible.” (Workshop 4, Patient 5) “Yes, but you always try to do that.” (Workshop 4, Patient 2)	Patients delay seeking care until symptoms become severe	Uncertainty during initial symptoms	Lack of trustworthy guidance
You get left alone in another way if I’m going to compare when I came in with a heart attack or with COPD. Because then you get to lie down… and you get medicine, of course. But you are alone long moments. (Workshop 1, Patient 5)	COPD patients feel left alone in hospital	Feeling abandoned	Increased vulnerability during hospitalisation
What makes a staff member good or not good? (Workshop 4, Interviewer 1) They listen and take in what you say. They don’t judge you directly. (Workshop 4, Patient 1) Yes, you get the feeling that they know what it’s all about. (Workshop 4, Patient 5) And that they take it seriously. (Workshop 4, Patient 2) Yes, exactly. (Workshop 4, Patient 5) Being taken seriously and everything. (Workshop 4, Patient 2)	Respectful treatment is important for patients	Desire for respectful encounters	Increased vulnerability during hospitalisation
And then … at least I did… felt now the last time I came home and you’re alone, that you recognize everything… You’re looking… but wondering, the feces, was it good this morning? Or was it something…? And what’s the colour of urine? And why is this itchy? Thing that you don’t worry about normally. But then, all of a sudden, you feel everything. Only that makes you worry unnecessarily. (Workshop 1, Patient 5)	Suddenly patients worry a lot after discharge	Need for post-discharge support	Discharge issues

## Results

The analysis resulted in experiences and expectations that encompass four categories: lack of trustworthy guidance, increased vulnerability during hospitalisation, early discharge issues, and advocacy for COPD recognition. An overview of these categories and their respective subcategories is presented in Table 5.

**Table 5:** Overview of categories and subcategories

Categories	Subcategories
Lack of trustworthy guidance	• Uncertainty during initial symptoms • Expectation of encountering knowledgeable staff • Need for improved communication
Increased vulnerability during hospitalisation	• Feeling abandoned • Desire for respectful encounters • Appreciation of familiarity and continuity of care
Discharge issues	• Feeling unprepared and unsupported • Need for post-discharge support
Advocacy for COPD recognition	• Struggles with resource shortages • Lack of supportive tools for COPD exacerbation • Desire for public recognition of COPD

### Lack of trustworthy guidance

This category delves into participants’ perspectives on accessing trustworthy guidance for their conditions. It includes their experiences and the support they seek when COPD symptoms initially manifest, highlighting the significance of knowledgeable healthcare practitioners during hospitalisation, and exploring patients’ visions for disease related information access and communication.

#### Uncertainty during initial symptoms

Feelings of uncertainty when deciding whether and when to seek help from healthcare practitioners were highlighted by the participants. They expressed a sense of insecurity in assessing their own condition independently and making informed decisions. Some noticed that warning symptoms sometimes improved spontaneously, while other times they worsened, complicating the decision to seek healthcare. Overall, participants expressed a reluctance towards hospital visits, citing the energy drain associated with each visit. Many denied needing help, especially when newly diagnosed, and tried to *“hold out as long as possible”* (Workshop 2, Patient 3) when the initial symptoms appeared. This often led to delayed visits and worsened conditions, causing frustration for not seeking help sooner.

*“I find it difficult to decide when I really need to seek help. I always end up doing it too late because I believe in self-healing. So I always wait too long.”* (Workshop 2, Patient 3).

To address these delays, participants expressed a need for trustworthy guidance on promptly reacting to initial symptoms including knowing whom to contact and when. Early assistance was stressed as vital for receiving optimal care.

#### Expectation of encountering knowledgeable staff

Participants highlighted the significance of knowledgeable healthcare practitioners for trustworthy guidance during COPD exacerbations, especially as initial points of contact. Many relied on calling 1177 as their initial step when experiencing symptoms, especially those living distant from hospitals who used it to avoid unnecessary trips. While 1177 often provided relief, some participants encountered responders who are registered nurses lacking specific COPD expertise, undermining their trust.

*“It’s a bit different depending on who you talk to when calling 1177 for help. Sometimes I get vague answers*,* making me a bit angry because I don’t call if I feel good or pretty good. I call when I feel bad. It wouldn’t occur to me otherwise. I get a bit angry. I know best.”* (Workshop 3, Patient 1).

Furthermore, participants noted insufficient COPD knowledge among emergency department healthcare practitioners, hindering appropriate responses to COPD-specific queries. They noted that emergency department healthcare practitioners had limited autonomy, resulting in prolonged treatment times due to the need for multiple consultations. Participants strongly desired a designated contact person, preferably from the lung department, to address their initial symptom inquiries.

#### Need for improved communication

Improving information access and enhancing communication during hospitalisation were seen as crucial for establishing a coordinated routine and motivating patient engagement. Participants felt more comfortable asking questions when they were thoroughly informed by healthcare practitioners. However, they often received fragmented information due to insufficient collaboration among healthcare practitioners, highlighting the importance of improving interprofessional communication.

*“(They said) ‘We’re going to cut down on the oxygen and we’re going to…’ and I feel like I got really bad information there all the time. And sometimes the doctor had signed a note before the nurse knew about it*,* and that’s what I thought was very annoying.”* (Workshop 1, Patient 2).

Participants expressed a preference for easily understandable information delivered at appropriate times. They preferred not to engage with healthcare practitioners during severe episodes when intense symptoms made communication difficult.

*“If it’s towards the end when I’m ready to go home*,* then it can be really nice to meet people. But the first day when you are so bad that you just want to scream at the first one.”* (Workshop 3, Patient 5).

Opinions on digital guidance were mixed. While participants recognised the benefits of social media platforms like Facebook for knowledge sharing and peer support, they recognised that not everyone could navigate digital technologies easily. They also valued educational websites for COPD but preferred concise resources, such as short pamphlets with essential information, over extensive website content.

### Increased vulnerability during hospitalisation

This category captures the circumstances in which patients felt most vulnerable due to their compromised physical and mental health conditions while hospitalised. It outlines the factors that heightened their vulnerability and elucidates their preferences for how they wanted to be treated in such situations.

#### Feeling abandoned

Participants commonly felt abandoned amidst the bustling environment of the high-volume hospital, recognising the demanding nature of healthcare practitioners’ workload. Extended periods of waiting alone at the emergency department intensified their anxiety, even verging on death agony, which led to the reluctance to undergo an emergency department visit. Furthermore, after initial interventions at the emergency department improved patients’ acute symptoms, they often experienced a sense of being overlooked or forgotten when left alone again. Participants expressed a need for healthcare practitioners to actively check in and provide reassurance during their stay at the emergency department, ensuring they feel noticed and supported throughout their care journey.

*“In the emergency room*,* when healthcare practitioners walk past the room. I wish they could at least open the door and say “-We haven’t forgotten about you”.”* (Workshop 3, Relative).

*“Yes*,* sometimes you do wonder.”* (Workshop 3, Patient 5).

*“Yes. You [her husband] lie there in the examination room*,* all quiet and everything*,* and you [her husband] hear people running outside. Well*,* what about me [her husband]? What about me [her husband]? Just that you [her husband] feel that someone is checking in on you [her husband].”* (Workshop 3, Relative).

Given their past negative experiences at the emergency department, participants sometimes opted for ambulance transport to avoid prolonged waiting periods and to seek more compassionate interactions with healthcare practitioners. They valued the immediate care and attention provided by healthcare practitioners at the emergency department, who consistently monitored and attended to their needs upon arrival via ambulance.

#### Desire for respectful encounters

Participants felt disrespected and unheard by healthcare practitioners during hospitalisation, hindering effective communication. They emphasised the importance of staff attitude and behaviour in shaping their experience, often feeling their words were not taken seriously. For instance, they recalled situations where ambulance staff prioritised monitoring oxygen levels over listening to the patient’s or the relative’s concerns. Participants felt compelled to deliberately demonstrate the severity of their condition to persuade ambulance staff that further care was necessary.

*“They [ambulance staff] often come and say that you should inhale*,* and then you feel better. And then they [ambulance staff] say “-Now your oxygenation is good”. But I don’t think at all I’m good enough. I’ve always started by saying*,* “-Before you leave*,* I want to go to the toilet”. And then I go*,* and when I get halfway to the toilet*,* they say*,* “-Wait*,* we’ll help you”. Then they [ambulance staff] will take me to the hospital.”* (Workshop 3, Patient 1).

Similarly, interactions with 1177 phone service staff sometimes involved questioning patients’ experiences, exacerbating feelings of disrespect, especially during symptoms like breathing difficulties.

*“Now you can go in and look at the medical record too*,* because the providers take notes of what you have said during the conversations. I called 1177 and said*,* ‘-I have severe COPD*,* I’m having a lot of trouble breathing*,* and I can hardly breathe.’ Then I saw a note that says*,* ‘She has mild respiratory problems. The recommendation is that she goes to a health centre tomorrow’*,* which is awful.”* (Workshop 3, Patient 1).

Additionally, participants felt disrespected due to the lack of information provided by healthcare practitioners about examinations and discharge procedures at the hospital, leaving them feeling ignored and vulnerable. This highlighted the need for clearer communication from healthcare practitioners.

#### Appreciation of familiarity and continuity of care

Responding to inquiries from healthcare practitioners while in a compromised condition was described as overwhelming for participants. Their poor condition often hindered their ability to engage in conversation and interact with unfamiliar faces. They preferred being recognised as known patients upon hospital arrival.

*“For me*,* the most crucial aspect is not having to retell my entire story every time I encounter a new staff who isn’t familiar with my medical history. What truly matters is feeling secure and understood—that sense of safety is the most important thing.”* (Workshop 2, Patient 2).

The presence of COPD nurses, who were approachable and familiar with their medical history, was greatly appreciated. Participants found comfort in the personalised and targeted advice provided by these nurses.

*“But I am fortunate to have a COPD nurse who knows me very well. And she knows*,* I don’t call unless I need to.”* (Workshop 1, Patient 2).

However, participants also admitted to feeling overly reliant on COPD nurses. Despite understanding the nurses’ heavy workload, they felt desperate when their symptoms flared up and required immediate attention from the nurse.

A sense of comfort was expressed when healthcare practitioners who had previously treated them followed up with them after discharge from hospital, emphasising the strong connection and familiarity. This continuity of care made them feel safe and secure, knowing that they were receiving ongoing support from familiar faces.

### Discharge issues

Being discharged early was commonly reported, often leaving participants feeling emotionally unsupported and lacking crucial information. This category underscores the challenges they faced during and after early discharge, detailing their expectations regarding discharge procedures and the support they anticipated at home.

#### Feeling unprepared and unsupported

Participants often found their health had not improved post-discharge compared to when they initially left their home. Early discharge, especially when living alone, heightened anxiety about health and mortality.

*“When you’ve been so sick and felt so close to death*,* the struggle to breathe is a terrible feeling. Coming home alone after that*,* you notice every little thing. You look around*,* wondering*,* was the bowel movement okay this morning? What about the color of urine? Why is this itching? These are things you wouldn’t normally worry about*,* but suddenly*,* you’re hyper-aware of everything. It’s just that it makes you unnecessarily anxious.”* (Workshop 1, Patient 5).

Receiving insufficient information in the discharge situation compounded their anxiety. They stressed the importance of being well-informed about discharge instructions when their condition allowed effective communication. However, premature discharge often left them with limited time and energy to engage fully in the discharge planning process. They expected more tailored information to alleviate their anxiety and emphasised the importance of receiving this information as early as possible to provide reassurance and facilitate a smoother transition out of the hospital.

Participants expressed frustration with healthcare practitioners who often overlooked their home environments when giving discharge advice, feeling that their specific living situations warranted personalised guidance to facilitate easier adherence. Those living alone struggled to follow discharge instructions, feeling unsupported and unable to manage their care effectively.

*“This summer has been completely disgusting. I came home alone*,* having no one home*,* it’s just me. Then you are even more insecure and even more afraid.”* (Workshop 3, Patient 5).

#### Need for post-discharge support

Participants were often reluctant to leave the hospital due to insufficient home support. Only a few received satisfactory home care, particularly appreciating the geriatric and palliative home care teams for their personalised and on-call support.

*“They [palliative team] are truly amazing. When I feel anxious and call them*,* even at five o’clock in the evening saying*,* ‘-I’m not feeling well’*,* they responded immediately*,* arriving at my house just half an hour later. It gives me such incredible peace of mind and security.”* (Workshop 3, Patient 4).

*“It was last autumn when I was really bad and was told all of a sudden that ‘-well*,* now you’re going home’. And I was alone*,* and then they contacted the geriatric centre. And they came to my home*,* they called me*,* they contacted me. They cared every single day. It was a huge help.”* (Workshop 3, Patient 5).

They also stressed the importance of involving family members in the care process post-discharge, recognising their crucial role in providing support and navigating through symptoms and care procedures. However, participants often felt guilty about burdening their family members during hospitalisation.

*“Now I almost start to cry*,* my husband had a really hard time. We had a hard time in two different ways. He had a hell when I became ill*,* to get me to hospital and take care of me when I got home.”* (Workshop 1, Patient 1).

While participants saw potential in digital solutions enabling remote meetings with healthcare practitioners at home, they stressed their opposition to relying solely on digital support without human interaction, underscoring the importance of personal connections in healthcare interactions.

### Advocacy for COPD recognition

This category encompasses participants’ encounters with inadequate resources in the care they received, their views on possible remedies, and the lack of social attention they faced, concretising their need for organisational support.

#### Struggles with resource shortages

Participants faced shortages of resources, including healthcare practitioners and hospital beds, which impacted their experiences of healthcare. They recounted being transferred to different departments post-emergency department, due to limited bed availability in the lung department. This unpredictability left participants feeling uncertain, with some likening the department assignment to *“a lottery winning ticket”* (Workshop 3, Patient 1) rather than being based on specific criteria. Transitioning between departments added to the challenges for participants, causing exhaustion and stress for both patients and healthcare practitioners.

*“Within the two days I was hospitalised I had to move to three different departments. But I think the staff suffered more than I did.”* (Workshop 1, Patient 2).

*“Yes*,* I noticed that the staff just can’t be nice [due to stress].”* (Workshop 1, Patient 5).

Those receiving care in other departments than the lung department after the triage at the emergency department felt neglected because the assistance provided was not tailored to meet their needs. Participants recognised that healthcare practitioners in these departments might not possess specialised knowledge of COPD, contributing to their dissatisfaction with the care they received. Despite their frustrations, participants empathised with the heavy burden borne by healthcare practitioners, especially during the demanding period of the COVID-19 pandemic when resources were stretched thin.

*“I feel conflicted because I know that hospital staff*,* including nurses and some doctors*,* are very kind and genuinely want us to get better. But they often lack the necessary resources to provide the level of care they aspire to*,* and I find that tragic.”* (Workshop 1, Patient 5).

#### Lack of supportive tools for COPD exacerbations

Since participants acknowledged the limitations of resources in healthcare, they emphasised the importance of utilising supportive tools to streamline the coordination of their care during COPD exacerbations. They noted a disparity in healthcare contacts between COPD exacerbations and events like heart attacks, despite similar perceived risks. Participants expressed feeling a greater sense of compassion and support during heart attack episodes compared to COPD exacerbations.

*“During the heart attack*,* I was never left alone*,* with things happening quickly. Doctors and nurses accompanied me beyond just transporting me to surgery. It’s a completely different experience coming in with a heart attack compared to COPD deterioration.”* (Workshop 3, Patient 5).

The lack of established protocols or routines compounded the complexity and uncertainty surrounding COPD exacerbations. Consequently, participants expressed a desire for supportive tools to enhance their care routine, such as discharge checklists at the emergency department and hospital wards, as well as a checklist for 1177 services. They believed that implementing these tools could improve care coordination and mitigate challenges posed by resource shortages to some extent.

#### Desire for public recognition of COPD

Participants emphasised the importance of public recognition and policymaker involvement in improving COPD exacerbation care. They suggested that policymakers should gain firsthand experience by visiting hospitals to better understand healthcare challenges and patient needs, potentially leading to improved policies and services.

*“Policymakers need to go out and visit the hospital ward sometimes*,* or at least see how it is done*,* and not just sit and make decisions that they don’t really have a real idea of what it’s all about.”* (Workshop 2, Patient 5).

Moreover, participants noted a widespread lack of social awareness surrounding COPD, leading to feelings of shame and stigma associated with the diagnosis, as one participant described “you feel that you have yourself to blame” (Workshop 3, Patient 1). This stigma hindered participants’ confidence in actively engaging in their condition management. They advocated for informational activities involving well-known individuals to raise awareness about COPD and combat stigma.

## Discussion

Our study contributes to understanding the experiences of individuals with severe COPD regarding hospital care and reveals some deficiencies, particularly in admission procedures, information exchange, interactions with healthcare practitioners, and transitions from hospital to home. Hospital care experiences extend beyond the inpatient stay, reflecting the complex and interconnected nature of care for people with severe COPD, consistent with recent research highlighting similar findings [29]. Additionally, participants in this study emphasised the positive impact of care continuity, supported by COPD nurses and home care teams, on their experience of exacerbations. When assessing the overall quality of COPD care, it is critical to consider the quality of hospital care, especially for those with severe COPD who are frequently hospitalised. However, the full spectrum of the hospitalisation experience for these individuals remains insufficiently studied [10]. Our study helps in addressing this gap in the literature, providing valuable insights that can inform future improvements in COPD hospital care.

The study emphasises the critical need for greater awareness and recognition of COPD, especially within the ‘advocacy for COPD recognition’ category. This echoes concerns from the GOLD Board of Directors about insufficient attention from global health entities [30]. Consistent with findings in studies from other parts of the world [31, 32], participants in our study highlighted widespread ignorance about COPD, extending to various facets of limited resources experienced in COPD exacerbation care, despite the significant economic burden COPD imposes in high-income countries [33–35]. Participants likened their experience during COPD exacerbations to that during a heart attack, expressing concern over perceived disparities in attention received. While interventions for heart attacks have seen substantial advancements due to their recognised severity [36, 37], COPD has not received comparable attention, reflecting our initial observation of early discharge as a systemic problem. However, the one-year mortality risk after an exacerbation of COPD is higher than that after an heart attack [38], highlighting the significance of managing COPD exacerbations. Insufficient resources in COPD care can lead to premature discharges, resulting in mistrust of the healthcare system, emotional distress, vulnerability, and potentially increased rehospitalisation rates. Negative experiences, such as anxiety in connection to hospital care, may contribute to mistrust in the care process. This can result in the overutilisation of some certain medical resources, such as using ambulance services in non-essential situations, as ambulances allowed patients to avoid waiting in emergency rooms where they often felt forgotten. Given the substantial financial burden the healthcare system already faces in treating COPD hospitalisations [39], it is imperative to minimise unnecessary expenditures. However, in addition to cutting extraneous costs, it is equally important to secure funding for essential interventions [33]. Addressing the resource deficiencies in COPD care requires policymakers to understand that investing in effective COPD interventions is not a financial burden but rather a potential source of significant economic returns in the near future [33]. Greater transparency regarding projected COPD-related healthcare costs can influence decision-making and facilitate better provision of COPD care at the management level, providing essential data for effective resource allocation [33]. Beyond relying solely on secondary data for decision-making, our findings indicate the value of decision-makers engaging in direct visits to hospitals. This hands-on approach allows them to gain valuable insights into the realities of the situations they seek to improve.

Participants specifically highlighted the critical issue of staff shortages in COPD care, in particular the lack of COPD nurses. While nurse-centred care is recognised for its importance [12], a shortage of COPD nurses is problematic since patients depend on direct contact with their nurses. Our study also pointed out vulnerabilities in a COPD care system that relies too much on COPD nurses, burdening them with excessive responsibilities. Increased responsibility for nurses to compensate for insufficient routines or COPD competence in other professions has also been pointed out in previous studies [40, 41]. To address this, interventions such as advanced data management tools have been suggested to improve workflow and staff efficiency [42]. Implementing further strategies to balance the workload of nurses is vital.

Lack of understanding and persistent misconceptions about COPD may prevent patients from accessing strategies to improve their lives [43]. Research highlights that increased disease knowledge is important for enhancing care quality among people with COPD [44–46]. The result from the present study complements these findings by identifying specific gaps in patient education related to the hospitalisation process and highlighting the preferred methods through which patients expect to receive this knowledge. Our study underscores the importance of adequate patient knowledge in managing initial exacerbation symptoms, which greatly improves overall care quality. In this regard, digital technologies present a promising avenue for delivering timely, highly customised information tailored to individual needs, along with fostering peer support and interaction, which has proven especially crucial during the COVID-19 pandemic [47, 48]. However, patients have expressed concerns that current digital educational tools lack sufficient personalisation, diminishing their willingness to engage. This observation is consistent with existing literature, which underscores the importance of personalised education for COPD patients, who often face multiple comorbidities. Without this tailored approach, patients can become overwhelmed, limiting the effectiveness and acceptance of educational interventions [45]. Furthermore, our study identifies varying levels of digital literacy among patients as a potential threat to health equity, necessitating efforts to address these barriers, for instance, developing user-friendly interfaces to lower entry barriers of technology use can facilitate greater access and usability [47, 49].

Educating healthcare practitioners, particularly those providing initial assistance during COPD exacerbations, such as staff at 1177 and emergency departments, is equally important, as emphasised in the subcategory ‘expectation of encountering knowledgeable staff’. Similar knowledge gaps exist among primary care providers, who diagnose and monitor most COPD cases [50]. Such knowledge gaps impede effective collaboration among healthcare practitioners, which is crucial for delivering high-quality COPD care [51, 52]. However, given the current fragmentation of the healthcare system, expecting all hospital staff to possess specialised COPD expertise, as suggested by the patients, is unrealistic [41]. Considering common implementation barriers of healthcare practitioner education interventions, such as heavy workload and time constraints [53], participants recommended practical tools such as checklists to aid COPD care and foster interprofessional collaboration among healthcare teams involved in COPD hospital care. Additionally, our research in exacerbation settings aligns with previous studies conducted in other contexts [54], highlighting that stigma surrounding COPD can hinder effective management, underscoring the need for supportive environments. Therefore, integrating empathy education for healthcare practitioners may be a relevant contribution, as it could foster respectful encounters with patients by enhancing healthcare practitioners’ ability to listen, understand, and support patients [55].

Another important finding of the study is that it provides concrete examples of aspects within person-centred care during COPD hospitalisation [15, 56]. COPD care is complex since it includes managing multiple comorbidities and various contextual factors, which complicates guideline adherence [57]. Despite international recognition of its importance [58], our study illustrates the difficulties in providing person-centred care during COPD hospitalisation, which is similar to experiences with other types of acute hospitalisations [59]. This was highlighted by a lack of continuity of care in a familiar environment and a failure to consider the patient’s home situation when devising treatment plans. Patients expressed a greater need to be treated as familiar individuals when they were unwell, as they felt that communication barriers become more pronounced during illness. Our findings align with previous research highlighting the essential role of the COPD nurse in coordinating care [41, 51]. This study further accentuates the importance of these nurses in addressing initial symptoms, offering personalised advice, and maintaining continuity of care. Supporting previous research that links improved patient experiences to active involvement in discharge planning [60], our study highlights the necessity of developing treatment plans that actively engage patients and reflect their diverse living conditions and backgrounds to improve care quality. Our study also underscores the value of follow-up care from familiar healthcare practitioners, which patients find beneficial. Participants involved in geriatric and palliative home care particularly appreciated the ease of access and continuity these teams provided, enhancing their overall care experience. This highlights the pivotal role of palliative care in managing severe COPD exacerbations and improving care quality. The positive feedback supports the importance of integrating palliative care to enhance person-centred approaches and overall treatment outcomes, as indicated by prior research [61].

### Methodological discussion

In this study, we have taken several actions to strengthen the credibility, dependability, and transferability of our research, which are all key aspects of trustworthiness in qualitative research [28].

Credibility [28] was strengthened by including a diverse sample of patients and healthcare practitioners with varying perspectives, all deeply engaged in COPD hospital care. Given the complex and dynamic nature of hospital environments, particularly for advanced-stage patients, we considered co-creation to constitute a suitable research design. This method facilitated collaborative and equitable communication while actively involving patients in the research process, further enhancing the study’s credibility [16]. To ensure relevance while addressing potential recall bias, participants needed to have experienced at least one hospitalisation within six months before enrolment. To ensure that patients were not still suffering from an exacerbation, we included patients who had been stable for one month prior recruitment. While involving only five patients may limit full exploration of the inherently diverse experiences of this patient group, the richness of the data was enhanced by facilitating discussions with a family member, healthcare practitioners, a designer, and hospital managers through creative methods that encouraged interaction. In addition to their descriptions of patient experiences and expectations, the involvement of these stakeholders provided insights into organisational, clinical, and relational aspects of care that may shape and influence patient experiences. Additionally, a potential concern for credibility is that practitioners often lack COPD-specific knowledge as evidenced in the study finding of the subcategory ‘Expectation of encountering knowledgeable staff’. To address both credibility and perspectives from different professionals, we included a physician, nurse, physiotherapist, and dietician, all experienced in COPD care, for the co-creation workshops. This allowed patient-centred insights to be enriched by informed professional views. We also used visual materials to facilitate deeper interactions and extended the data collection process through home tasks arranged during the co-creation process, enhancing researchers’ understanding of the data and its overall richness. However, a potential limitation of co-creation is that power dynamics may discourage patients from challenging healthcare practitioners or sharing honest opinions, affecting data credibility [62]. To address this potential bias, we ensured that patients were represented in equal or greater numbers than any other individual stakeholder group, both in the overall co-creator group and within each subgroup discussion. We also used creative methods to help reduce power imbalances among participants. Despite the many benefits of co-creation, it must be acknowledged that the in-person co-creation process is highly demanding for the patient population included in this study [62]. We addressed this issue by reducing the task burden during the planning of the workshops, which involved simplifying activities and providing ample break time. Co-creators expressed appreciation of the environment, as reported elsewhere [63]. Another potential source of bias is our introduction of early discharge at the beginning of the workshops and providing participants with a definition of what we considered to be ‘early’. While all patient participants had experienced early discharge based on their description, individual interpretations of what constitutes ‘early’ may vary and warrant exploration in future research. Importantly, discussions revealed broader issues that were of relevance to the patients, extending beyond hospital admission, stay, and discharge. We consider this an important finding of our study, underscoring hospital experiences inevitably connect to patients’ broader care journeys, influencing and being influenced by other care aspects.

Dependability refers to the consistency of data over time and adjustments made during analysis [28]. Continuous feedback through member checking ensured ongoing validation and refinement [16], enhancing the dependability of the findings. Additionally, iterative discussions within the research team were conducted throughout the analysis process to ensure dependability.

We made concerted efforts to enhance transferability [28, 64] by thoroughly documenting the study context, recruitment process, data collection, and analysis methods, as recommended in COREQ [18]. This transparency is a key feature of co-creation approaches as well and allows readers to evaluate whether the findings and co-creation process can be applied to other settings [16]. While the small sample size limits transferability, we adhered to the recommended co-creation group size of 10–12 participants to ensure meaningful interaction [16]. This aligns with the principle of information power, which suggests that the more relevant information a sample holds for the study, the fewer participants are needed [65]. Our diverse group of 13 participants, with repeated contributions and recurring patterns that emerged in later workshops, provided sufficient depth. Although only one family member was included and a single viewpoint clearly cannot represent all caregiver experiences, it nevertheless offered valuable insights that complemented and balanced those of patients and healthcare practitioners in the co-creation process. A larger follow-up study could further validate and broaden these findings.

## Conclusions

This is, to our knowledge, one of the first studies to explore patients’ experiences and expectations of the whole process around hospitalisations due to ECOPD. The study provides valuable insights into the integration of person-centred care in managing COPD exacerbations. By exploring the nuanced experiences and expectations of people with COPD, it generates knowledge essential for establishing routines for hospital care that better align with patient needs. Key areas for suggested interventions include promoting proactive help-seeking during initial symptoms, enhancing patient education, providing specialised training for healthcare practitioners on COPD, ensuring continuity of care, improving discharge services, and raising public awareness. Moreover, our research emphasises that each patient’s hospitalisation experience and expectations are shaped by individual circumstances, underscoring the importance of addressing person-centred care in the design of these interventions. Promoting person-centred care during hospitalisations involves delivering care in familiar environments to minimise communication barriers, actively engaging patients in collaborative discharge planning, and seamlessly integrating home care teams into the overall treatment process. Ultimately, these findings contribute to the groundwork for improving the quality of hospital care.

## Supplementary Information

Supplementary Material 1.

## Data Availability

The datasets used and/or analysed during the current study are available from the corresponding author.

## Abbreviations

COPD: Chronic Obstructive Pulmonary Disease
ECOPD: Exacerbation of Chronic Obstructive Pulmonary Disease
GOLD: Global Initiative for Obstructive Lung Disease
FEV1: Forced Expiratory Volume in One Second
COREQ: Consolidated Criteria for Reporting Qualitative Research
