# Supplementary material for: A qualitative study of patient experiences and expectations around hospital care during exacerbations of chronic obstructive pulmonary disease – a health CASCADE study
Source: BMC Pulm Med. 2025 Nov 13;25:524. doi: 10.1186/s12890-025-04024-x (PMC12616904; doi:10.1186/s12890-025-04024-x)
Supplement: Supplementary file 1 — Supplementary Material 1. [file 12890_2025_4024_MOESM1_ESM.docx]

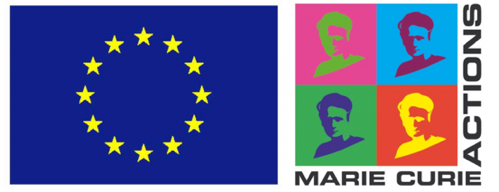

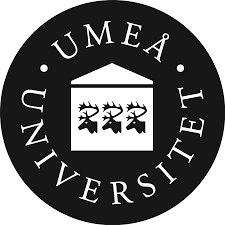

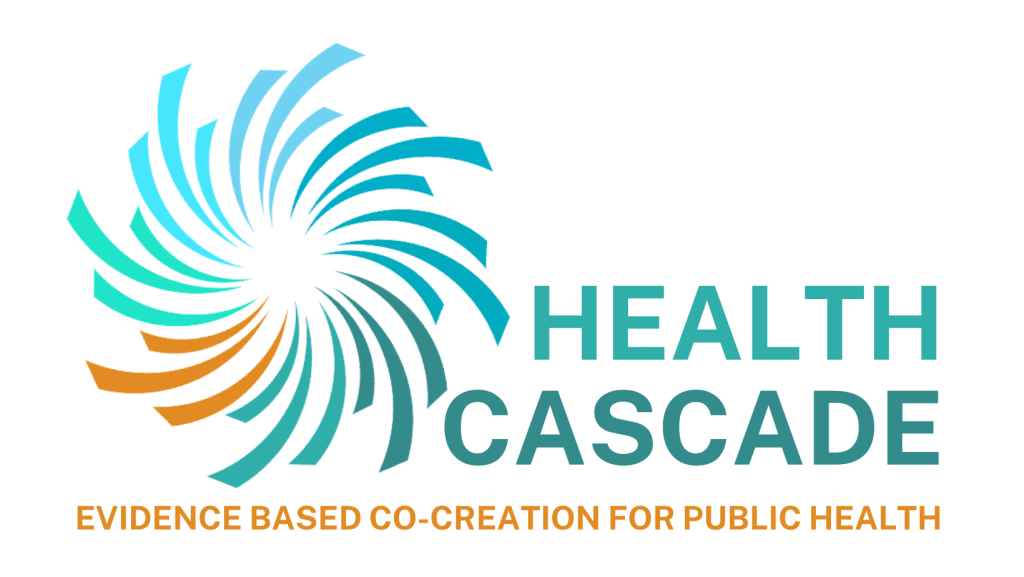


Supplemental file 1

“During the heart attack, I was never left alone”

A qualitative study of experiences and expectations around hospital care during acute exacerbations of chronic obstructive pulmonary disease

Question guides

# Workshop 1

## Question guide for three subgroup discussions

1. What are the reasons people with COPD are not treated sufficiently before they are discharged from hospital?

- Consider the following dimensions:
  - Politics: How do healthcare policies and regulations contribute to the issue of early discharge for people with COPD?
  - Society: What societal factors lead to insufficient hospital care for people with COPD?
  - Environment: In what ways do hospital environments and community settings affect the care provided to people with COPD?
  - Technology: What are the technological limitations or advancements that influence the discharge related interventions for people with COPD?
  - Economy: How do economic factors, such as funding and resource allocation, impact the early discharge of people with COPD?

**2. What are the consequences of people with COPD not being treated sufficiently before they are discharged from hospital?**

- Consider how early discharge affects the following stakeholders:
- **Patients:** How does early discharge affect their recovery and long-term health?
- **Physicians:** What challenges do physicians face as a result of early discharge of people with COPD?
- **COPD nurses:** How does early discharge influence the coordination and continuity of care by COPD nurses?
- **Managers:** What are the managerial implications of early discharge practices?
- **Physiotherapists:** How are physiotherapists affected in terms of treatment planning and patient outcomes due to early discharge?
- **Family members:** How does early discharge affect the family members who support and care for people with COPD?

# Workshop 2

## **Task 1: question guide for three subgroup discussions**

**1. How would you like hospital care for COPD exacerbations to work in the best of worlds?**

- Describe the ideal scenario for handling COPD exacerbations within hospitals.

## **Task 2: question guide for the card game (five pairs)**

**Figure 1 illustrates an example of a translated version of the templates utilised in the card game. Figure 2 showcases a fully completed card game template.**

**1.What would your imagined life in the hospital and primary care center look like if the problems are solved?**

- Include dimensions such as:
  - **People:** Who are involved and how do they interact?
  - **Task:** What tasks are performed and how are they managed?
  - **Technology:** What technologies are used and how do they facilitate care?
  - **Structure:** How is the care system organised and what processes are in place?
  - Interaction objects: How would you like to interact with commonly seen objects at the hospital?

**2. What would your imagined life at home look like if the problems are solved?**

- Include dimensions such as:
  - **People:** Who are involved in home care and what are their roles?
  - **Task:** What tasks are necessary for maintaining health and well-being at home?
  - **Technology:** What technologies support home care and patient monitoring?
  - **Structure:** How is home care organised and coordinated?
  - Interaction objects: How would you like to interact with commonly seen objects at the hospital?

**3. What would your imagined life in the community look like if the problems are solved?**

- Include dimensions such as:
  - **People:** Who do you interact with and how does the community support you?
  - **Task:** What activities and tasks are part of your daily life **in the community**?
  - **Technology:** What technologies facilitate your life **in the community**?
  - **Structure:** How is the support system **in the community** organised and managed?
  - Interaction objects: How would you like to interact with commonly seen objects at the hospital?


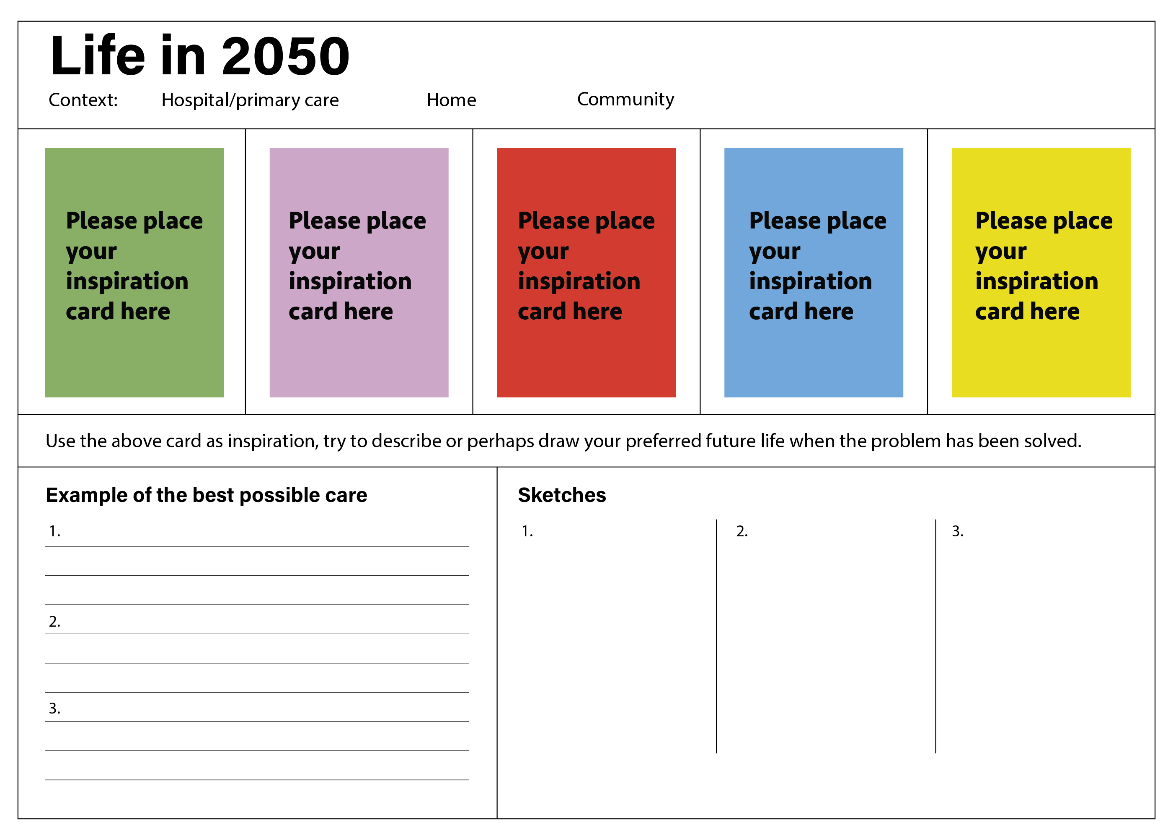


**Figure 1. Template for the card game used in the second workshop (translated version). Coloured rectangles indicate where participants placed cards: green for technology, purple for people, red for communication, blue for interaction objects, and yellow for tasks. White cards can be created by the players and are not restricted to predefined dimensions**.


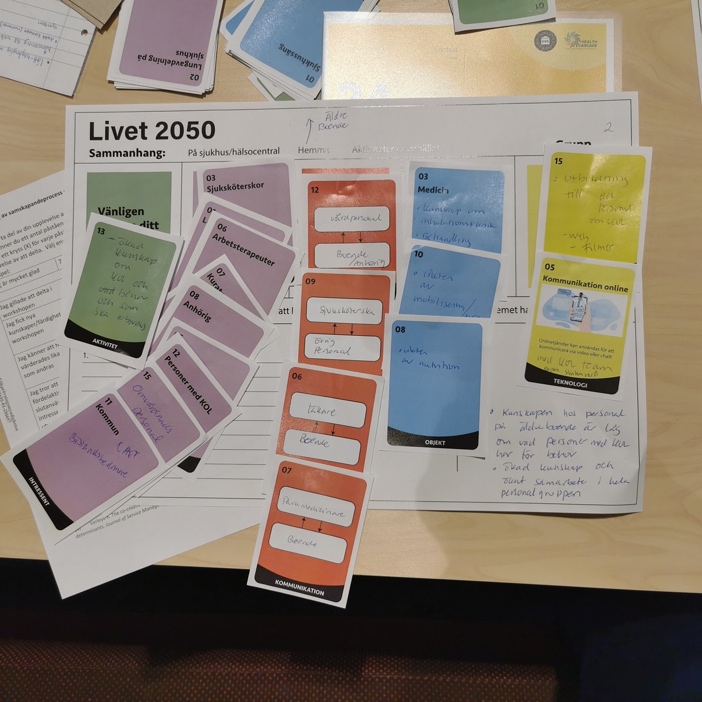


**Figure 2. Example of a completed template after the workshop.**

# Workshop 3

Specific stages tailored to the hospitalisation process for people with COPD include being in a stable disease phase, symptoms appearing, calling for help, the way to the hospital, emergency department, waiting room, examinations, lung department/other department due to filled capacity of the lung department, meeting physicians and nurses, examinations, interventions, treatment from other healthcare practitioner groups, discharge, home, care received from primary care and municipality, and follow-up provided by healthcare practitioners. Figure 3 illustrates the template for this process, while Figure 4 displays a completed version of the template.

## Question guide for the patient group

**1. Please add the options or additional steps when experiencing the service.**

- Detail any additional steps or choices available to patients during their service experience.

**2. Use one word or sentence to express your emotional journey at each step.**

- Describe the emotional impact at various stages of the service experience.

**3. Add your pain point to each step and a potential solution to it.**

- Identify specific pain points at each stage and suggest potential solutions to address them.


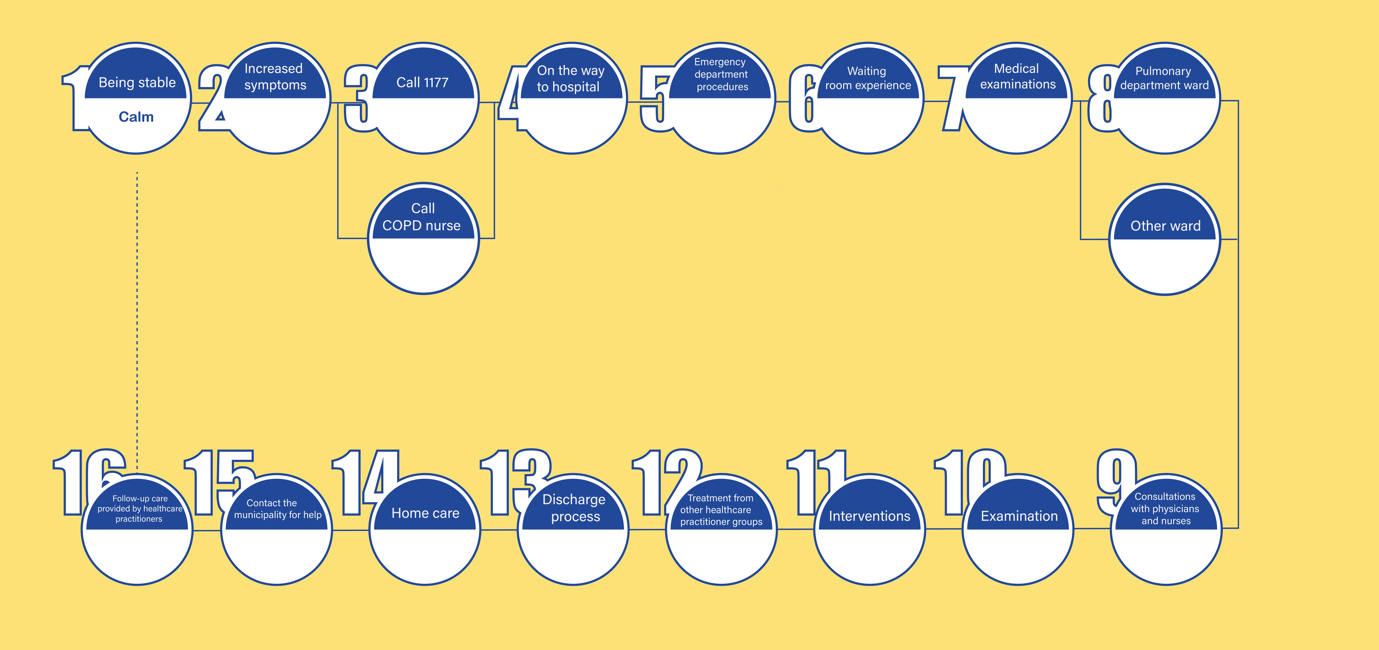


**Figure 3. Template designed for the patient journey map (translated version). 1. being stable, 2. increased symptoms, 3. seeking help, 4. on the way to hospital, 5. emergency department procedures, 6. waiting room experience, 7. medical examinations, 8. medical ward, 9. consultations with physicians and nurses, 10. examination, 11. interventions, 12. treatment from other healthcare practitioner groups, 13. discharge process, 14. home care, and 15. contact the municipality for help, 16. follow-up care provided by healthcare providers.**


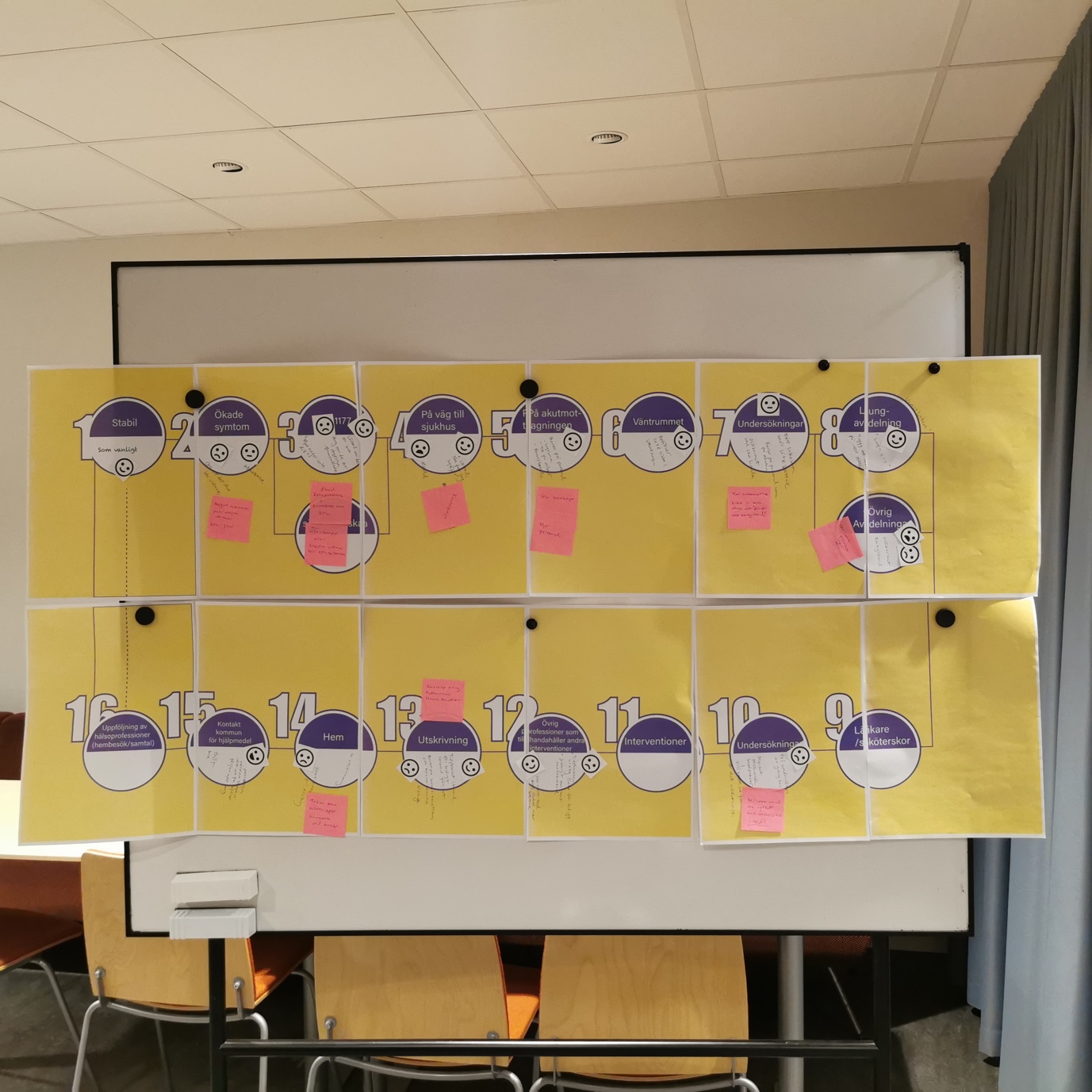


**Figure 4. The completed template after the workshop.**
